# 17β-Estradiol Attenuates Neuropathic Pain Caused by Spared Nerve Injury by Upregulating CIC-3 in the Dorsal Root Ganglion of Ovariectomized Rats

**DOI:** 10.3389/fnins.2019.01205

**Published:** 2019-11-08

**Authors:** Zhen-Zhen Xu, Qin-Yi Chen, Shi-Yu Deng, Meng Zhang, Chao-Yang Tan, Yang Wang, Ke-Tao Ma, Li Li, Jun-Qiang Si, Li-Cang Zhu

**Affiliations:** ^1^Department of Anesthesiology, First Affiliated Hospital of Shihezi University, Shihezi, China; ^2^Department of Physiology, Shihezi University School of Medicine, Shihezi, China; ^3^Key Laboratory of Xinjiang Endemic and Ethnic Disease, Shihezi University School of Medicine, Shihezi, China; ^4^Department of Anesthesiology, Xiangyang Central Hospital, Hubei University of Arts and Science, Xiangyang, China; ^5^Department of Anesthesiology, Sichuan Academy of Medical Sciences, Sichuan Provincial People’s Hospital, Chengdu, China; ^6^Department of Physiology, Medical College of Jiaxing University, Jiaxing, China; ^7^Department of Physiology, School of Basic Medicine and Tongji Medical College, Huazhong University of Science and Technology, Wuhan, China; ^8^Department of Physiology, School of Basic Medical Sciences, Wuhan University School of Medicine, Wuhan, China

**Keywords:** 17β-estradiol, ClC-3, spared nerve injury, neuropathic pain, ovariectomy

## Abstract

17β-estradiol plays a role in pain sensitivity, analgesic drug efficacy, and neuropathic pain prevalence, but the underlying mechanisms remain unclear. Here, we investigated whether voltage-gated chloride channel-3 (ClC-3) impacts the effects of 17β-estradiol (E2) on spared nerve injury (SNI)-induced neuropathic pain in ovariectomized (OVX) female Sprague Dawley rats that were divided into OVX, OVX + SNI, OVX + SNI + E2, OVX + SNI + E2 + DMSO (vehicle, dimethyl sulfoxide), or OVX + SNI + E2+Cltx (ClC-3-blocker chlorotoxin) groups. Changes in ClC-3 protein expression were monitored by western blot analysis. Behavioral testing used the paw withdrawal threshold to acetone irritation and paw withdrawal thermal latency (PWTL) to thermal stimulation. Immunofluorescence indicated the localization and protein expression levels of ClC-3. OVX + SNI + E2 rats were subcutaneously injected with 17β-estradiol once daily for 7 days; a sheathed tube was implanted, and chlorotoxin was injected for 4 days. Intrathecal Cltx to OVX and OVX + SNI rats was administered for 4 consecutive days (days 7–10 after SNI) to further determine the contribution of ClC-3 to neuropathic pain. Patch clamp technology in current clamp mode was used to measure the current threshold (rheobase) dorsal root ganglion (DRG) neurons and the minimal current that evoked action potentials (APs) as excitability parameters. The mean number of APs at double-strength rheobase verified neuronal excitability. There was no difference in behaviors and ClC-3 expression after OVX. Compared with OVX + SNI rats, OVX + SNI + E2 rats showed a lower paw withdrawal threshold to the acetone stimulus, but the PWTL was not significantly different, indicating increased sensitivity to cold but not to thermal pain. Co-immunofluorescent data revealed that ClC-3 was mainly distributed in A- and C-type nociceptive neurons, especially in medium/small-sized neurons. 17β-estradiol administration was associated with increased expression of ClC-3. 17β-estradiol-induced increase in ClC-3 expression was blocked by co-administration of Cltx. Cltx causes hyperalgesia and decreased expression of ClC-3 in OVX rats. Patch clamp results suggested that 17β-estradiol attenuated the excitability of neurons induced by SNI by up-regulating the expression of ClC-3 in the DRG of OVX rats. 17β-estradiol administration significantly improved cold allodynia thresholds in OVX rats with SNI. The mechanism for this decreased sensitivity may be related to the upregulation of ClC-3 expression in the DRG.

## Introduction

Neuropathic pain, a form of allodynia or hyperalgesic spontaneous pain, remains a major challenge for pain researchers and clinicians (Fukuda et al., 2017; Xu et al., 2017; Zhang et al., 2018; Ouyang et al., 2019). Inflammatory-mediator release at the site of injury triggers alterations in the properties of primary afferent neurons and increases their excitability leading to ectopic, stimulus-independent activity (Colloca et al., 2017; Alles and Smith, 2018). Changes in ion channels are responsible for development of abnormal discharge (Amescua-Garcia et al., 2018; Xu et al., 2018). Recent research has shown that intracellular Cl^–^ concentration in DRG neurons increased after sciatic nerve section or inflammation (Funk et al., 2008; Si et al., 2019). Studies have focused on chloride channels in primary sensory neurons (PSN), as activation of chloride channels in sensory neurons may cause chloride efflux and depolarization because of high intracellular chloride concentrations (Mao et al., 2012; Bonin and De Koninck, 2013). Numerous studies have shown that anion channels, and particularly chloride channels, may be involved in the pathogenesis of neuropathic pain (Wang L.-J. et al., 2017; Si et al., 2019). Actually, downregulation of ClC-3 in DRG neurons contributes to mechanical hypersensitivity following peripheral nerve injury (Riazanski et al., 2011). Thus, modulation of ClC-3 function may be a novel therapeutic avenue for the treatment of neuropathic pain (Pang et al., 2016). Many studies examining the pathogenesis of neuropathic pain as well as its prevention and treatment strategies have suggested that the pain threshold is sex-specific (Ramirez-Barrantes et al., 2016; Vacca et al., 2016). Estrogen receptors are distributed in many pain-related regions in the central and peripheral nervous systems, and 17β-estradiol can affect the generation and transmission of pain on many levels (Amandusson and Blomqvist, 2013). It has been reported that estrogen has a palliative effect on neuropathic pain, but the underlying mechanisms are complex (Lu et al., 2013; Ma et al., 2016; Lee et al., 2018). Estrogen can activate ClC-3 via ERα in the cell membrane of osteoblasts (Deng Z. et al., 2017), promote proliferation of ER^+^ breast cancer MCF-7 cells through the ClC-3 Cl^–^ channel pathway (Yang et al., 2018), and regulate ion channels in pain modulation, but its effects on analgesia and promotion of pain are inconsistent (Berman et al., 2017; Ren et al., 2018). Numerous studies have reported that estrogen can provide pain relief in females (Vacca et al., 2016; Lee et al., 2018). However, there have been no systematic studies on the effects of estrogen replacement therapy on neuropathic pain in menopausal women. The present work aimed to identify whether ClC-3 plays a role in the effects of estrogen on neuropathic pain in ovariectomized (OVX) rats.

## Materials and Methods

### Animals

Adult female Sprague Dawley rats (10–12 weeks old, 200–250 g, *n* = 180) were purchased from the Animal Center of the Xinjiang Medical University (Ürümqi, China). Animal use was approved by the Committee of Animal Experimental Ethics of the First Affiliated Hospital of Medical College, Shihezi University, China. Animals were housed in plastic boxes with controlled temperature (24 ± 2°C), humidity (40–50%), and a 12:12 h light:dark cycle. We selected rats with relatively uniform and stable baseline responses to cold and hot stimuli for the experiment. Rats were OVX bilaterally, and the sham OVX (ShamOVX) group underwent operations as previously described (Chen et al., 2018; Chang et al., 2019). All protocols were approved by the Animal Ethics Committee of the First Affiliated Hospital of Shihezi University School of Medicine (approval No. A2018-165-01) on February 26, 2018, and were consistent with the Guidelines for the Care and Use of Laboratory Animals, published by the United States National Institutes of Health.

### Surgical Procedure to Induce a Neuropathic Pain Model by Spared Nerve Injury

We used SNI to prepare a model of neuropathic pain as previously reported (Xu et al., 2017). Experimental procedures were performed on animals under anesthesia with sodium pentobarbital (40 mg/kg, intraperitoneal, Sigma-Aldrich, St. Louis, MO, United States). Care was exercised to prevent infection and reduce the impact of inflammation. After the skin was cut, the sciatic nerve and its three terminal branches were exposed directly through the part formed by the biceps muscle: the lateral side, common fibular nerve, and tibial nerves. The tibial and common peroneal nerves were cut and ligated by SNI, and the sural nerve was preserved. As the common peroneal and tibial nerves are closely connected, followed by removing the distal nerve ends about 3–5 mm. Care was taken not to damage the nearby sural nerve. After surgery, all wounds were irrigated with sterile saline and closed in layers.

### Groups and Drug Intervention

All OVX rats were randomly divided into five groups: OVX, OVX + SNI, OVX + SNI + estradiol (E2), OVX + SNI + E2 + DMSO, and OVX + SNI + E2 + chlorotoxin (Cltx). For intrathecal delivery (1 μM/L, 20 μl/day, Sigma-Aldrich) (Thompson and Sontheimer, 2016), Cltx was dissolved in 30% DMSO and injected through a catheter for 4 days. Intrathecal catheters were implanted on SNI day 7 as previously described (Pogatzki et al., 2000). Briefly, a sterile catheter filled with saline was inserted through the intervertebral space at L_5_/L_6_, and the tip of the tube was positioned at the lumbosacral spinal level. Animals with hindlimb paralysis or paresis after surgery were excluded. Animals without movement disorders received lidocaine (2%) through the catheter to verify the intraspinal location. Immediate bilateral hindlimb paralysis (within 15 s) lasting 20–30 min confirmed correct catheterization. Animals without these features were excluded from subsequent experiments. DRGs for patch clamps were incubated with Cltx *in vitro*. The 7-day procedure of 17β-estradiol (30 μg/kg/day, subcutaneous, Sigma-Aldrich) administration was performed as previously described (Vacca et al., 2016).

### Measurement of Serum 17β-Estradiol Levels

Rats were deprived of food overnight, and serum 17β-estradiol levels were assessed according to a previously described protocol (Homberg et al., 2018). Briefly, blood samples were collected from the abdominal aorta under anesthesia, and serum was separated by centrifugation at 15,000 r for 5 min. Serum corticosterone levels were measured with a corticosterone enzyme immunoassay kit (Cayman Chemical, Ann Arbor, ML, United States). Analyses were conducted in duplicate. The intra-assay coefficients of variation were lower than 10% for each analysis.

### Behavioral Assays

#### Heat Hyperalgesia (Hot Plate Test)

Thermal hyperalgesia was assessed according to a previously described protocol (Ouyang et al., 2019; Si et al., 2019). The thermal withdrawal latency in response to radiant heat stimulation was measured with an analgesia meter (Ugo Basile, Stoelting, IL, United States). Animals were placed in the chamber and allowed to acclimatize for 30 min before testing. A radiant heat source was focused under the glass floor beneath the hind paws. Thermal-stimulus intensity was adjusted to obtain a baseline thermal withdrawal latency of approximately 20 s. The digital timer automatically recorded the duration between stimulus initiation and thermal withdrawal latency, and a 30 s cutoff was used to prevent tissue damage. Each rat was tested every 5 min, and the average of six trials was used as the PWTL.

#### Cold Allodynia (Acetone Drop Method)

Cold sensitivity was measured by applying a drop of acetone to the plantar surface of the hind paw as previously described (Deng et al., 2015; Bergeson et al., 2016). Rats were housed and habituated for 30 min in transparent plastic boxes with a wire-mesh floor. After the adaptation period, acetone was gently applied against the plantar skin of the left hind paws with an acetone bubble formed with a 0.1-ml syringe, alternately three times to hind paw at intervals of 5 min, and the duration of licking or biting and remaining in the air was recorded. Each rat was tested every 5 min, and the average of six trials was used as the PWCL.

### Sample Preparation

At the predetermined time points, the animals were deeply anesthetized with sodium pentobarbital (40 mg/kg, intraperitoneal; Sigma). Rats were sacrificed after behavioral testing was performed, and ipsilateral L_4__–__6_ DRGs tissues were collected. Samples for RT polymerase chain reaction (RT-PCR) and western blot experiments were snap-frozen in liquid nitrogen and stored at −80°C. Samples used for immunofluorescence imaging were perfused through the ascending aorta with saline, followed by 4% paraformaldehyde in 0.1 M phosphate buffer (4°C, pH 7.4) as previously reported (Zhang et al., 2017).

### Immunofluorescence

The L_4__–__6_ DRG on the surgical side was removed and fixed in 4% paraformaldehyde overnight, followed by dehydration in 20% or 30% sucrose in phosphate buffer at 4°C. The tissue was cut into 5-μm thick sections with a cryostat (Leica CM1950, Nußloch, Germany). The sections were blocked with 20% bovine serum albumin (BSA) for 1 h in a 37°C incubator (303-0S; Beijing Ever Bright Medical Treatment Instrument Co., Ltd., Beijing, China), washed with phosphate-buffered saline (PBS), and incubated with primary antibody (rabbit anti-ClC-3 polyclonal antibody; 1:100, 13359S, CST) overnight at 4°C. After washing with PBS, the sections were incubated with secondary antibody (TRITC-conjugated anti-rabbit secondary antibody; 1:100; Santa Cruz Biotechnology, Heidelberg, Germany) for 1 h at 37°C. For double immunofluorescence staining, tissue sections were incubated with a mixture of anti-ClC-3 antibody and antibodies against neurofilament-200 (NF-200; a marker for myelinated A-fibers, 1:100; ab82259; Abcam, Cambridge, United Kingdom), calcitonin gene related peptide (CGRP, a marker of peptidergic C-type neurons, 1:100; ab81887; Abcam) for 2 nights at 4°C, or IB4 (FITC-conjugated; a marker for non-peptidergic C-type neurons, 5 μg/ml; L2895; Sigma). Except for IB4-treated tissue sections, the other sections were treated with a mixture of FITC- and TRITC-conjugated secondary antibodies at a 1:100 dilution for 1 h at 37°C. IB4 was 1:750 mixed with TRITC-conjugated secondary antibody. The sections were rinsed with 0.01 M PBS three times, mounted on gelatin-coated slides, and air dried. Immunoreactivity was visualized by fluorescence microscopy, and a negative control was used by omission of the primary antibody to confirm the specificity of the immunoreaction. Sections were observed at 200× magnification using a confocal laser scanning microscope (LSM710; Carl Zeiss AG, Oberkochen, Germany). Optical density measurements and data analysis of CLCN3-positive cells for the two types of DRG neurons were performed using Image-Pro Plus 6.0 (Media Cybernetics, Rockville, MD, United States). The percentage fluorescence results of positive neurons of three independent experiments were recorded.

### Western Blot Analysis

Frozen tissues were homogenized, and proteins were extracted using a nucleoprotein and cytoplasmic protein extraction kit (Keygen Biotech, Nanjing, China) and 30 μg of protein was mixed with sodium dodecyl sulfate sample buffer. Proteins were separated on standard sodium dodecyl sulfate-polyacrylamide gel electrophoresis (8–10% gels) and transferred onto 0.45-μm nitrocellulose membranes (Invitrogen, Carlsbad, CA, United States). Membranes were blocked in 5% milk for 1 h and incubated overnight at 4°C with the following primary antibodies: mouse anti-ClC-3 (1:750 dilution; ab134285; Abcam) and anti-β-actin (1:1000 dilution, ab8226, Abcam). The next day, the membranes were rinsed with *tris*-buffered saline Twenty three times for 10 min and incubated with the secondary antibodies (anti-mouse immunoglobulin G against the primary antibodies). Staining was visualized using enhanced chemiluminescence (GE Healthcare, Chicago, IL, United States). Band intensities were quantified by ImageJ software (Rawak Software Inc., Germany).

### Quantitative RT-PCR Analysis

Total RNA was extracted from the ipsilateral L_4__–__6_ DRGs of rats using Trizol (Invitrogen) and reverse-transcribed to cDNA using a qRT-PCR kit (Invitrogen) according to the manufacturer’s instructions (Sang et al., 2018). For each cDNA target, 2 μL aliquots of each completed reverse transcriptase reaction were amplified in a 20 μL reaction volume using SYBR Green Real Time PCR Master Mix (Toyobo Co., Ltd., Osaka, Japan) in 45 cycles of 95°C and 60°C for 12 s and 35 s, respectively. The following primers were used for amplification: ClC-3, 5′-ATGCTTGGTCAGGATGGCTTGTAG-3′ (forward) and 5′-AGT CATCCAGTCAGCAGCAATGTC-3′ (reverse); β-action, 5′-AGCAGA TGT GGATCAGCAAG-3′ (forward) and 5′-AACAGTCCGCCTAGAAGCAT-3′ (reverse). We used the mRNA level of β-actin as an internal control, and we ran a standard curve to determine the relative levels of each cDNA target. Relative gene expression levels were calculated using the 2^–(ΔΔ*Ct*)^ method. The expression level of each gene was analyzed in triplicate.

### Isolation of DRG Neurons

L_4__–__6_ DRG neurons from the ipsilateral side of the operation were dissociated using enzyme digestion as previously described (Zhang et al., 2017). The drug-intervention group DRGs were treated with 17β-estradiol and Cltx. Briefly, the excised ganglia were freed from their connective tissue sheaths and cut into pieces with a pair of sclerotic scissors in DMEM/F12 medium (GIBCO; Thermo Fisher Scientific, Waltham, MA, United States) under low temperature on ice. The fragments were transferred into 5 mL of DMEM/F12 medium containing trypsin (0.4 mg/mL, Sigma) and collagenase (type IA, 0.6 mg/mL, Sigma) and incubated for 5 min at 37°C. The ganglia were then gently triturated using fine fire-polished Pasteur pipettes. The suspension was dissociated in DMEM/F12 medium, supplemented with 10% fetal bovine serum, and DRG neurons were plated on glass cover slips coated with Poly-L-Lysine (Sigma). Cells were maintained in a humidified atmosphere (5% CO_2_, 37°C) and used for electrophysiological recordings 6–24 h after plating.

### Electrophysiological Recordings

All recordings were performed on small and medium diameter (20–35 μm) neurons as previously described (Chen et al., 2011). Coverslips with DRG neurons were mounted in a small flow-through chamber positioned on the stage of an inverted microscope (Nikon Eclipse Ti, Tokyo, Japan) to select DRG cells with smooth membrane surfaces and good translucency for experiments. Coverslips were continuously perfused with gravity-driven bath solution. Standard whole-cell patch-clamp recordings from isolated DRG neurons were performed at room temperature (22°C) using an EPC-10 amplifier and the PULSE program (HKA Electronics, Lambrecht, Germany). The membrane capacitance was read from the amplifier by PULSE to measure the size of cells and current densities. Glass pipettes (3–5 MΩ) were prepared with a Sutter P-87 puller (Sutter Instruments, Novato, CA, United States). Action potentials were elicited by a series of depolarizing currents from 0 to 500 pA (150 ms) in 50-pA step increments under the current clamp mode to measure the current threshold (rheobase) in the vicinity of the explosive action potential current. The current was altered by 10 pA per step, i.e., the minimal current that evoked an action potential, as a parameter for excitability. The recorded signal was amplified by a MultiClamp 700B amplifier (Molecular Devices, LLC, Sunnyvale, CA, United States), filtered at 10 kHz, and converted by an Axon Digidata 1550A D/A converter (Molecular Devices) at a sampling frequency of 10 to 20 kHz. Voltage errors were minimized by using 80–90% series resistance compensation, and linear leak subtraction was used for all recordings. For the current clamp experiments, the bath solution contained (in mM): 140 NaCl, 5 KCl, 2 CaCl_2_, 2 MgCl_2_, 10 D-glucose, 10 HEPES; the pH was adjusted to 7.4 with NaOH. The pipette solution contained (in mM): 30 KCl, 100 K-aspartate, 5 MgCl_2_, 2 Mg-ATP, 0.1 Na- GTP, 40 HEPES; the pH was adjusted to 7.2 with KOH. All chemicals were obtained from Sigma.

### Statistical Analysis

All data are expressed as mean ± SEM of three independent experiments. The normal distribution hypothesis of the test data and the homogeneity of variance were examined before further statistical analysis. Statistical analysis was performed using SPSS 10.0 (SPSS Inc., Chicago, IL, United States). PWCL and PWTL were analyzed using repeated-measures analysis of variance, and multiple comparisons between groups at each time point were conducted using Bonferroni’s *post hoc* tests. Regarding the western blot, PCR, and patch-clamp data, analysis among multiple groups was carried out by one-way analysis of variance followed by Tukey’s *post hoc* tests. Student’s *t*-test was used for two-group comparisons. *P* < 0.05 was considered statistically significant.

## Results

### The Established OVX Model Had No Effect on Cold and Thermal Hypersensitivity

**FIGURE 1:**
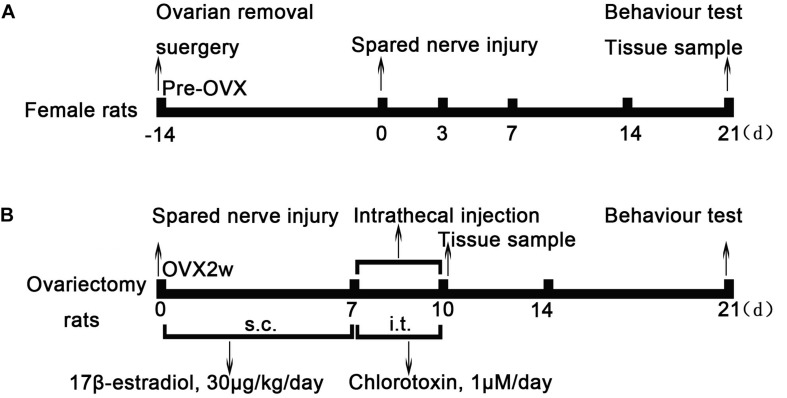
Experimental model and schedule of drug intervention. **(A)** Ovarian removal (OVX) and spared nerve injury (SNI) model protocol. Normal female rats underwent ovariectomy 2 weeks before SNI. Two weeks later, OVX rats underwent SNI and behavioral testing at different time points of SNI; the ipsilateral L_4__–__6_ dorsal root ganglion was obtained as tissue sample after behavioral testing. **(B)** After the SNI model was established, rats were treated with 17β-estradiol for 7 days (from day 0 to day 6, 30 μg/kg/day) subcutaneously. On the 7th day of SNI, intrathecal Cltx or DMSO (1 μM/day, 20 μL) was administered for 4 days. The L_4__–__6_ dorsal root ganglia of rats were collected on the 7th day and 10th day of SNI after behavioral testing. OVX, ovariectomy; SNI, spared nerve injury; E2, 17β-estradiol; Cltx, Chlorotoxin; DMSO, vehicle, dimethyl sulfoxide.

**FIGURE 2:**
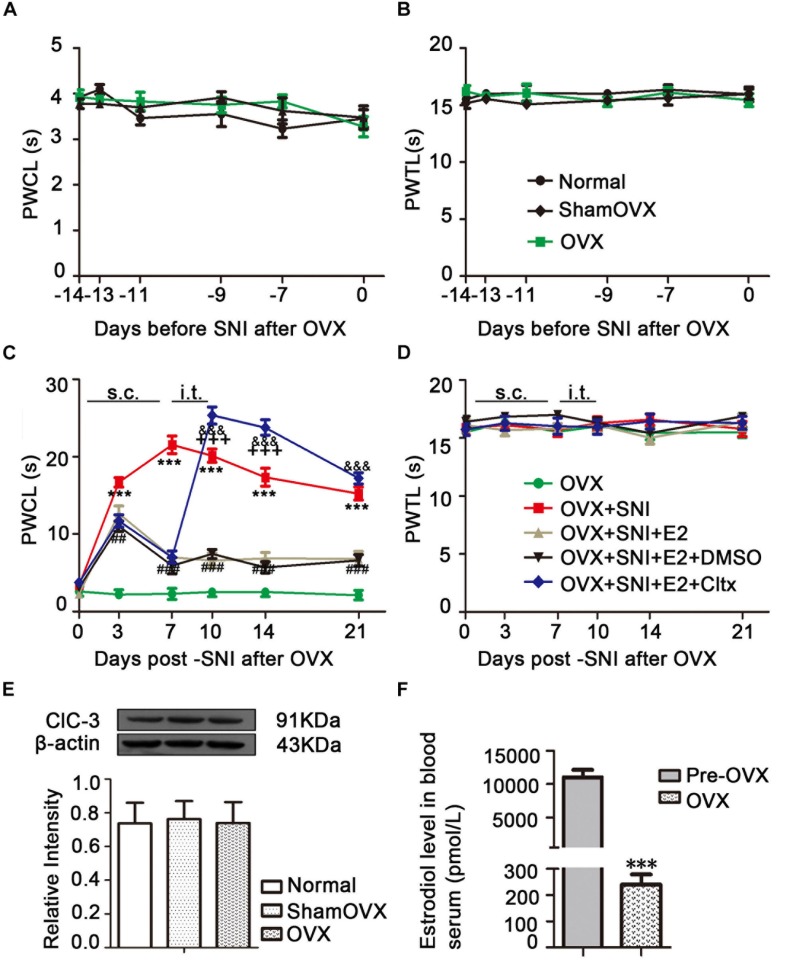
Effects of ovariectomy (OVX), spared nerve injury (SNI), and drug treatment on ClC-3 expression and behavior. **(A)** No change in the thermal threshold was observed after OVX (*n* = 8 per group). **(B)** There was no significant difference in duration of paw lifting in response to a cold stimulus (*n* = 9 per group). **(C)** Increased sensitivity to cold stimulation started from the 3rd day after SNI and lasted until the end of behavioral testing with slight recovery. Estrogen administration partially reversed this pain allergy in SNI rats until the day 21. Intrathecal injection of ClC-3 specific inhibitor, Cltx, resulted in cold hyperalgesia recovery. Vehicle solution had no effect (*n* = 6 per group). ^∗∗∗^*P* < 0.01, OVX + SNI vs. OVX group; ^##^*P* < 0.01, ^###^*P* < 0.001, OVX + SNI + E2 vs. OVX + SNI group; ^&⁣&⁣&^*P* < 0.001, OVX + SNI + E2 + Cltx vs. OVX + SNI + E2 group; ^+++^*P* < 0.001, OVX + SNI + E2 + Cltx vs. OVX + SNI group. **(D)** Thermal pain did not produce significant differences among all five groups. OVX, ovariectomy; SNI, spared nerve injury; PWTL, paw withdrawal thermal latency; PWCL, paw withdrawal cold latency; s.c., subcutaneous; i.t., intrathecal injection. **(E)** Western blot images of ClC-3 protein expression show that there were no significant differences after OVX; *n* = 6 per group. **(F)** Serum estrogen decreased significantly after ovariectomy; *n* = 6 per group), ^∗∗∗^*P* < 0.001, OVX vs. Pre-OVX.

Normal female rats underwent OVX 2 weeks before SNI (Figure 1A). Behavioral tests showed that the sensitivity to cold and heat stimulation had remained unchanged 2 weeks after OVX (Figures 2A,B), and ClC-3 expression in DRG neurons did not change significantly within these 2 weeks (Figure 2E). Estrogen levels were measured in rat blood samples collected from the abdominal aorta under anesthesia before and after ovarian resection. The results showed that 17β-estradiol levels were significantly lower in the OVX group compared to pre-OVX (Figure 2F; 11060 ± 1113 in the pre-OVX vs. 240.1 ± 38.07 in the OVX group, *P* < 0.001; *n* = 6 in each group).

### Development of Cold and Thermal Hypersensitivity After SNI Treatment in OVX Rats

An OVX + SNI model was used to stimulate neuropathic pain in menopausal female rats. These rats showed pain-sensitizing behaviors such as paw protection, paw licking, and dorsiflexion (data not shown). Behavioral tests showed that OVX + SNI rats developed significant cold hyperalgesia. The increased sensitivity to cold stimulation started on the 3rd day after SNI and lasted until the end of behavioral testing (Figure 2C and Supplementary Table S1; OVX + SNI group vs. OVX group on day 3, 16.70 ± 0.6117 vs. 2.215 ± 0.5856, *P* < 0.001; day 7, 21.53 ± 1.142 vs. 2.283 ± 0.7183, *P* < 0.001; day 10, 20.13 ± 0.8730 vs. 2.505 ± 0.5909, *P* < 0.001; day 14, 17.34 ± 1.156 vs. 2.503 ± 0.5914, *P* < 0.001; day 21, 15.24 ± 0.8483 vs. 2.117 ± 0.6256, *P* < 0.001; *n* = 6 in each group). There was no significant change in thermal stimulation (Figure 2D).

### ClC-3 Was Mainly Expressed in Medium/Small-Sized DRG Neurons of OVX Rats

**FIGURE 3:**
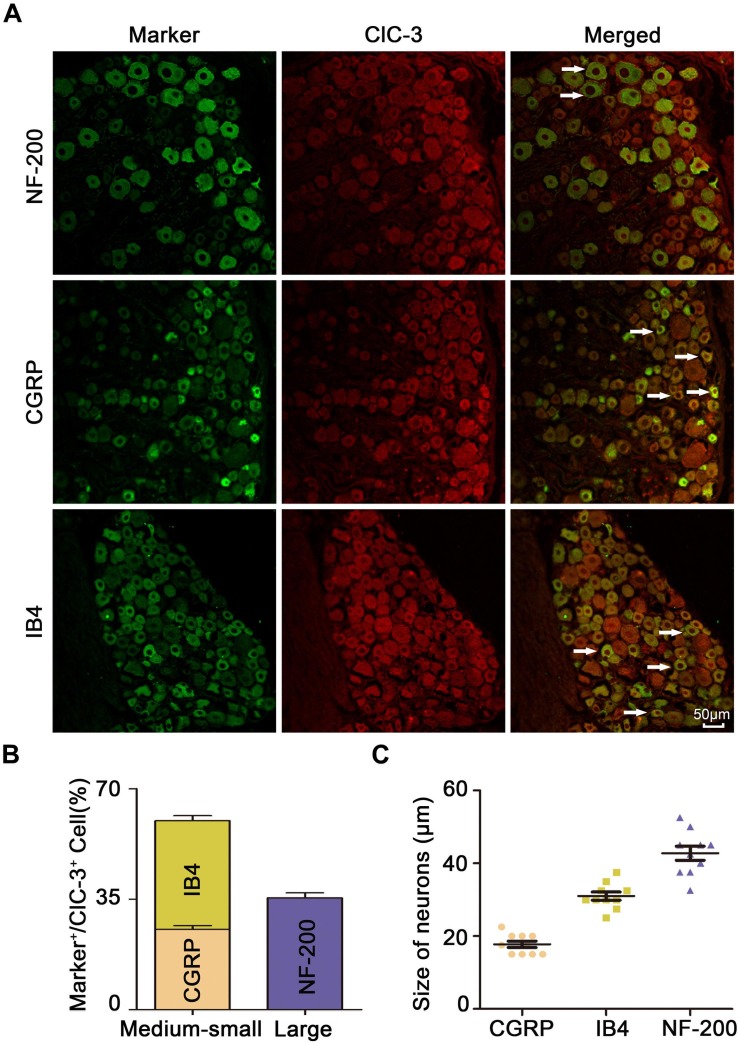
ClC-3 expression, mainly in medium/small-sized and in large DRG neurons. **(A)** Immunofluorescence double labeling revealed that the ClC-3 protein was colocalized with IB4 (a marker of non-peptidergic C-type neurons), CGRP (a marker of peptidergic C-type neurons), and NF-200 (a marker of A-type neurons). Arrows refer to co-labeled neurons, scale bar = 50 μm. **(B)** The percentage of IB4, CGRP, and NF-200 positive (green) neurons relative to ClC-3 (red) positive cells. **(C)** Neuronal diameter size of IB4, CGRP, and NF-200. DRG, dorsal root ganglion.

Immunofluorescent double staining experiments showed that ClC-3 protein colocalized with IB4, CGRP, and NF-200 (Figure 3A). The percentages of IB4-, CGRP-, and NF-200-positive neurons relative to the percentage of ClC-3-positive cells were 34.47 ± 1.602%, 25.43 ± 1.267%, and 35.41 ± 1.552%, respectively (*n* = 6 in each group; Figure 3B). These results showed that ClC-3 was mainly located in A- and C-type neurons in the DRG. The neuronal diameter size ranges of IB4, CGRP, and NF-200 were 31.00 ± 1.13, 17.75 ± 0.87, and 42.75 ± 1.917, respectively (Figure 3C; *n* = 10 in each group). ClC-3 expression, mainly in medium/small-sized as well as in large DRG neurons, indicated that ClC-3 may be involved in the regulation of superficial sensations such as pain.

### Downregulation of ClC-3 Expression in DRG Neurons After SNI in OVX Rats

**FIGURE 4:**
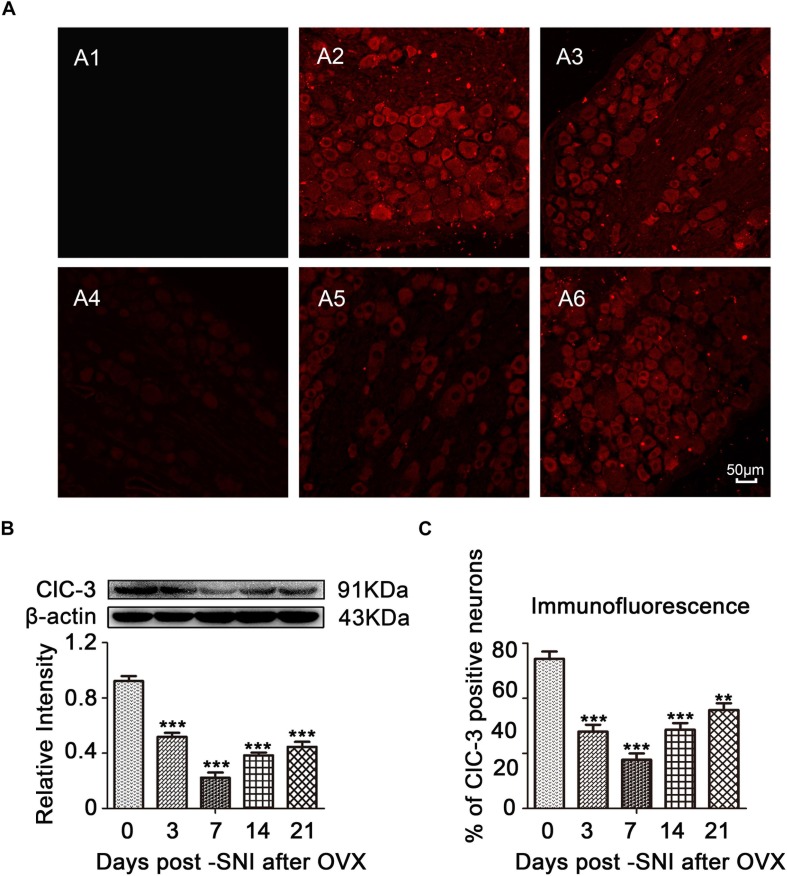
ClC-3 expression was decreased in a time-dependent manner in the ipsilateral L_4__–__6_ DRG neurons of SNI rats after OVX. **(A)** Immunofluorescent signal of ClC-3 (red) detected in the DRG neurons of SNI rats after OVX. A1: Negative control (PBS). A2: OVX. A3: OVX + SNI D3. A4: OVX + SNI D7. A5: OVX + SNI D14. A6: OVX + SNI D21. PBS, Phosphate buffered saline; D3, 3 days after SNI; D7, 7 days after SNI; D14, 14 days after SNI; D21, 21 days after SNI; scale bar = 50 μm. **(B)** Western blot analysis showed that the ClC-3 protein levels were altered in a time-dependent manner. A significant decrease was detected on day 7 after SNI; *n* = 6 per group, ^∗∗∗^*P* < 0.001, compared to OVX group. **(C)** Quantification of ClC-3 positive neurons in ipsilateral L_4__–__6_ DRGs of OVX and OVX + SNI rats; ^∗∗^*P* < 0.01, ^∗∗∗^*P* < 0.001, compared to the OVX group.

**FIGURE 5:**
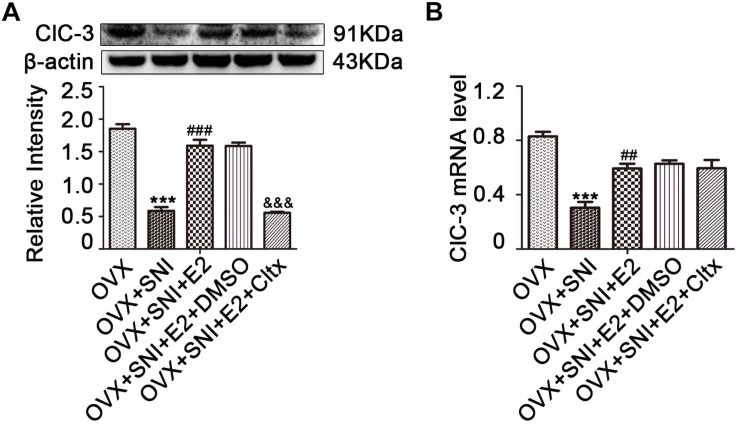
Cltx inhibited the 17β-estradiol mediated increase in ClC-3 protein expression but not in mRNA after intrathecal injection. **(A)** Subcutaneously injected 17β-estradiol (once per day for 7 consecutive days at SNI Day 0 to Day 6) reversed the downregulation of ClC-3 protein caused by SNI. Intrathecal injection of Cltx (once per day for 4 consecutive days starting at the 7th day of SNI after 17β-estradiol injection) reduced estrogen-mediated protein increased expression. Vehicle solution had no effect. *n* = 6 per group, ^∗∗∗^*P* < 0.001, OVX + SNI vs. OVX group; ^###^*P* < 0.001, OVX + SNI + E2 vs. OVX + SNI group; ^&⁣&⁣&^*P* < 0.001, OVX + SNI + E2 + Cltx vs. OVX + SNI + E2 group; E2: 17β-estradiol; Cltx, Chlorotoxin; D0, the day of SNI operation; D6, 6 days after SNI. **(B)** ClC-3 mRNA level decreased after SNI and was rescued by 17β-estradiol; repeated daily intrathecal injection of Cltx (1 μM) did not suppress *ClC-3* mRNA level. Vehicle solution had no significant effect; *n* = 6 per group. ^∗∗∗^*P* < 0.001, OVX + SNI vs. OVX group; ^##^*P* < 0.01, OVX + SNI + E2 vs. OVX + SNI group.

Immunofluorescent staining in rat ipsilateral L_4__–__6_ DRGs at different time points after SNI showed high distribution of ClC-3, and the positive cells in the ipsilateral DRGs decreased in a time-dependent manner after SNI (Figures 4A,C, 5A; OVX + SNI group vs. OVX group on day 3, 27.91 ± 2.528 vs. 54.34 ± 2.629, *P* < 0.01; day 7, 17.70 ± 2.350 vs. 54.34 ± 2.629, *P* < 0.001; day 14, 28.65 ± 2.378 vs. 54.34 ± 2.629, *P* < 0.001; day 21, 35.75 ± 2.485 vs. 54.34 ± 2.629, *P* < 0.01; *n* = 6 in each group). A significant change in ClC-3 protein was detected after SNI (Figures 4B, 5A). Quantification of ClC-3 protein by western blot analysis confirmed the time-dependent downregulation of ClC-3 protein in the DRG neurons, which was parallel to the time course of decrements in PWCL (OVX + SNI group vs. OVX group on day 3, 0.6483 ± 0.03598 vs. 1.153 ± 0.04463, *P* < 0.01; day 7, 0.2778 ± 0.04699 vs. 1.153 ± 0.04463, *P* < 0.001; day 10, 0.5855 ± 0.05903 vs. 1.853 ± 0.06955, *P* < 0.001; day 14, 0.4805 ± 0.02438 vs. 1.153 ± 0.04463, *P* < 0.001; day 21, 0.5570 ± 0.04517 vs. 1.153 ± 0.04463, *P* < 0.001; *n* = 6 in each group). These changes began on the 3rd day after SNI and reached the lowest point on day 7. The ClC-3 mRNA level was also downregulated on the 10th day after SNI (Figure 5B; OVX + SNI group vs. OVX group on day 10, 0.3800 ± 0.05292 vs. 1.037 ± 0.04256, *P* < 0.001; *n* = 6 in each group).

### 17β-Estradiol Administration Attenuated Cold Hyperalgesia in SNI OVX Rats

To evaluate the potential function of 17β-estradiol in neuropathic pain, it was subcutaneously injected once per day for 7 consecutive days, from day 0 to day 6 of SNI. In all SNI OVX rats that received 17β-estradiol (30 μg/kg/day), cold hyperalgesia was partially reversed, and the effect persisted from day 3 until the end of behavioral testing. For thermal hyperalgesia, the analgesic effect was not observed (Figure 2C; OVX + SNI + E2 group vs. OVX + SNI group on day 3, 12.67 ± 0.9605 vs. 16.70 ± 0.6117, *P* < 0.01; day 7, 7.017 ± 0.5443 vs. 21.53 ± 1.142, *P* < 0.001; day 10, 6.580 ± 0.9755 vs. 20.13 ± 0.8730, *P* < 0.001; day 14, 6.867 ± 0.7654 vs. 17.34 ± 1.156, *P* < 0.001; day 21, 6.807 ± 0.9490 vs. 15.24 ± 0.8483, *P* < 0.001; *n* = 6 in each group). The 17β-estradiol injection did not affect PWTL (Figure 2B).

### Restoration of ClC-3 Protein and mRNA Expression After 17β-Estradiol Administration

After 17β-estradiol administration, L_4__–__6_ DRG neurons were harvested on day 10 of SNI. ClC-3 protein and mRNA levels were measured and the results showed an increase in the expression level of ClC-3 protein (Figure 5A and Supplementary Figures S7–S12; on day 10 of SNI, OVX + SNI + E2 group vs. OVX + SNI group, 1.590 ± 0.09205 vs. 0.5855 ± 0.05903, *P* < 0.01; *n* = 6 in each group). The qRT-PCR results revealed that 17β-estradiol regulated the expression of ClC-3 at the mRNA level. The OVX + SNI + E2 group had higher ClC-3 mRNA levels compared to the OVX + SNI group (Figure 5B; on day 10 of SNI, OVX + SNI + E2 group vs. OVX + SNI group, 0.7420 ± 0.04419 vs. 0.3800 ± 0.05292, *P* < 0.01; *n* = 6 in each group).

### Intrathecal Cltx Administration Reproduced and Aggravated Hyperalgesia Relieved by 17β-Estradiol and Repressed ClC-3 Protein Level but Did Not Affect mRNA Upregulation by 17β-Estradiol

On the 7th day of SNI and consecutive administration of 17β-estradiol, Cltx (1 μM/day) or 10% DMSO as vehicle, 20 μL, was administered intrathecally to SNI rats for 4 consecutive days (Figure 1B; from day 7 to 10 after SNI). After receiving Cltx, cold hyperalgesia was restored (Figure 2C; on day 10 of SNI, OVX + SNI + E2 + Cltx vs. OVX + SNI + E2, 25.33 ± 1.113 vs. 7.427 ± 0.5994, *P* < 0.001; day 14, 23.77 ± 0.9978 vs. 5.700 ± 0.7425, *P* < 0.01; on day 10 of SNI, OVX + SNI + E2 + Cltx vs. OVX + SNI, 25.33 ± 1.113 vs. 20.13 ± 0.8730, *P* < 0.001, on day 14, 23.77 ± 0.9978 vs. 17.34 ± 1.156, *P* < 0.001; *n* = 6 in each group). Vehicle solution had no effect (*n* = 6 per group). L_4__–__6_ DRG tissues were harvested on day 10 of SNI after behavioral testing, and ClC-3 protein and mRNA levels were measured. Western blot analysis revealed that 17β-estradiol could not upregulate the expression of ClC-3 after Cltx was administered (Figure 5A; on day 10 of SNI, OVX + SNI + E2 + Cltx group vs. OVX + SNI + E2 group, 0.5563 ± 0.01588 vs. 1.590 ± 0.09205, *P* < 0.001; *n* = 6 in each group). The qRT-PCR and immunoblotting results were not consistent with the western blot analysis results, as Cltx administration did not regulate the expression of *ClC-3* mRNA (Figure 5B). Administration of vehicle solution had no effect on ClC-3 protein and mRNA expression.

### Intrathecal Cltx Administration in OVX and OVX + SNI Rats Increased Hyperalgesia and Downregulated ClC-3 Protein Expression

**FIGURE 6:**
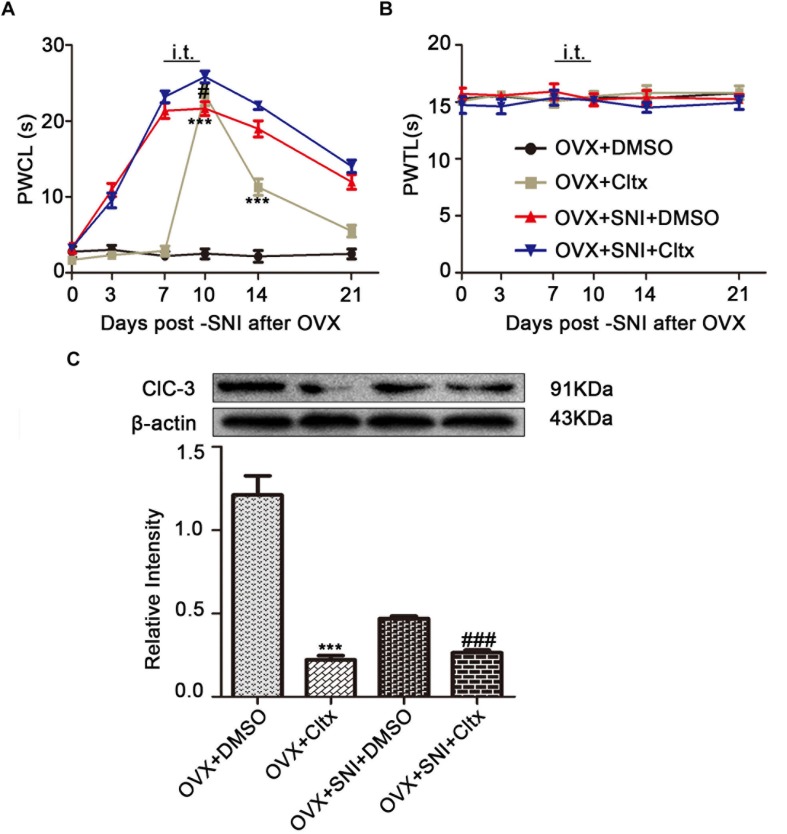
Intrathecal Cltx repressed ClC-3 protein expression and aroused cold hyperalgesia in OVX rats and aggravated hyperalgesia in OVX + SNI rats. **(A)** Repeated daily intrathecal injection of Cltx caused cold hyperalgesia in OVX rats from the injection day until the end of behavioral testing with slight recovery. Intrathecal injection of Cltx daily from SNI day 7 to day 10 aggravated hyperalgesia on day 10 of SNI (*n* = 6 per group). ^∗∗∗^*P* < 0.01, OVX + Cltx vs. OVX + DMSO group; ^#^*P* < 0.05, OVX + SNI + DMSO vs. OVX + SNI + Cltx group; DMSO: vehicle, dimethyl sulfoxide; PWCL, paw withdrawal cold latency; OVX, ovariectomized; SNI, spared nerve injury. **(B)** No change in the thermal threshold was observed (*n* = 6 per group). PWTL, paw withdrawal thermal latency. **(C)** Intrathecal injection of Cltx decreased ClC-3 protein expression both in OVX rats and OVX + SNI rats; *n* = 6 per group, ^∗∗∗^*P* < 0.001, OVX + Cltx vs. OVX + DMSO group; ^###^*P* < 0.001, OVX + SNI + DMSO vs. OVX + SNI + Cltx group.

To further determine the contribution of ClC-3 to neuropathic pain, Cltx 1 μM/day or 10% DMSO as vehicle, 20 μL, was administered intrathecally to OVX and OVX + SNI rats for 4 consecutive days (Days 7 to 10 after SNI for the OVX + SNI group, 2 weeks after OVX for the OVX group). Cold hyperalgesia appeared significantly altered in OVX rats on days 10 and 14 (Figure 6A and Supplementary Table S2; on SNI day 10, 2 weeks after OVX, OVX + Cltx vs. OVX + DMSO, 23.51 ± 1.489 vs. 2.505 ± 0.6632, *P* < 0.001; day 14, 11.28 ± 1.087 vs. 2.167 ± 0.7702, *P* < 0.01; on day 10 after SNI, OVX + SNI + Cltx vs. OVX + SNI + DMSO, 21.63 ± 0.9098 vs. 25.83 ± 0.7708, *P* < 0.05; *n* = 6 in each group). There was no significant change in thermal stimulation (Figure 6B). On day 10 of SNI and on SNI day 10, 2 weeks after OVX, Cltx was administered for 4 days and L_4__–__6_ DRG tissues were obtained after behavioral testing. Western blot analysis revealed that Cltx downregulated ClC-3 protein expression (Figure 6C and Supplementary Figures S1–S6; on day 10, OVX + Cltx vs. OVX + DMSO group, 0.1761 ± 0.02175 vs. 0.9674 ± 0.09262, *P* < 0.001; *n* = 6 in each group).

### 17β-Estradiol Decreased the Excitability of DRG Neurons Caused by SNI in OVX Rats When Blocked by Cltx

**FIGURE 7:**
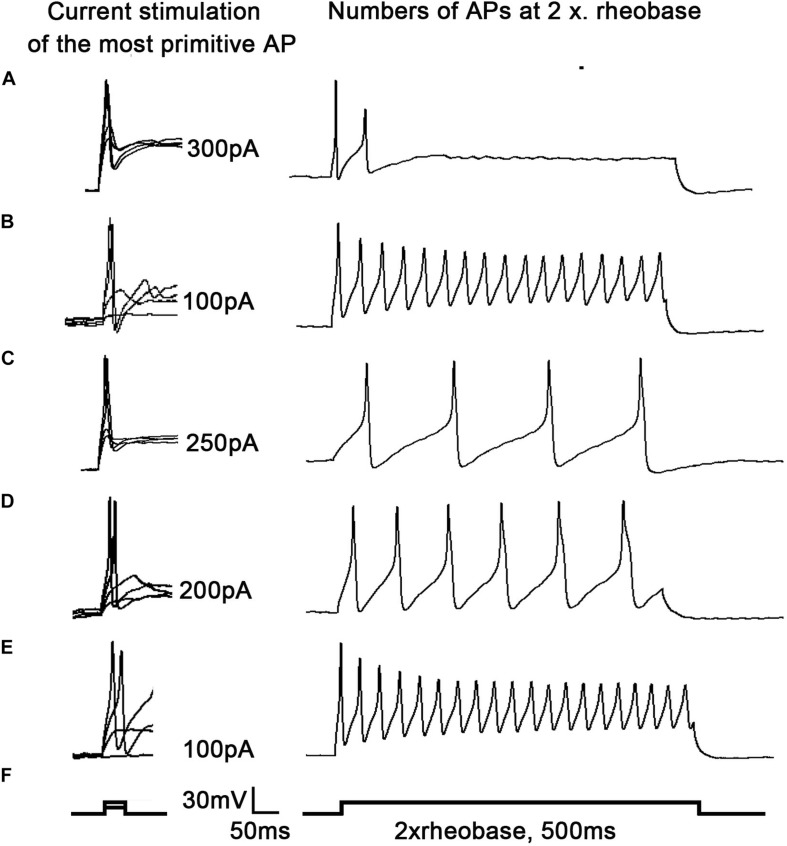
17β-estradiol attenuated increased excitability of DRG neurons in spared nerve injury ovariectomized rats and was inhibited by Cltx. Current threshold (rheobase) was determined as the current required for activating the first action potential. **(A–F)** On the right, representative traces of action potentials (APs) evoked by current injections into DRG neurons from OVX, OVX + SNI, OVX + SNI + E2, OVX + SNI + E2 + DMSO, and OVX + SNI + E2 + Cltx groups; *n* = 6 per group; On the right, twice in the figure, the number of action potentials produced at the corresponding 2 × rheobase.

**FIGURE 8:**
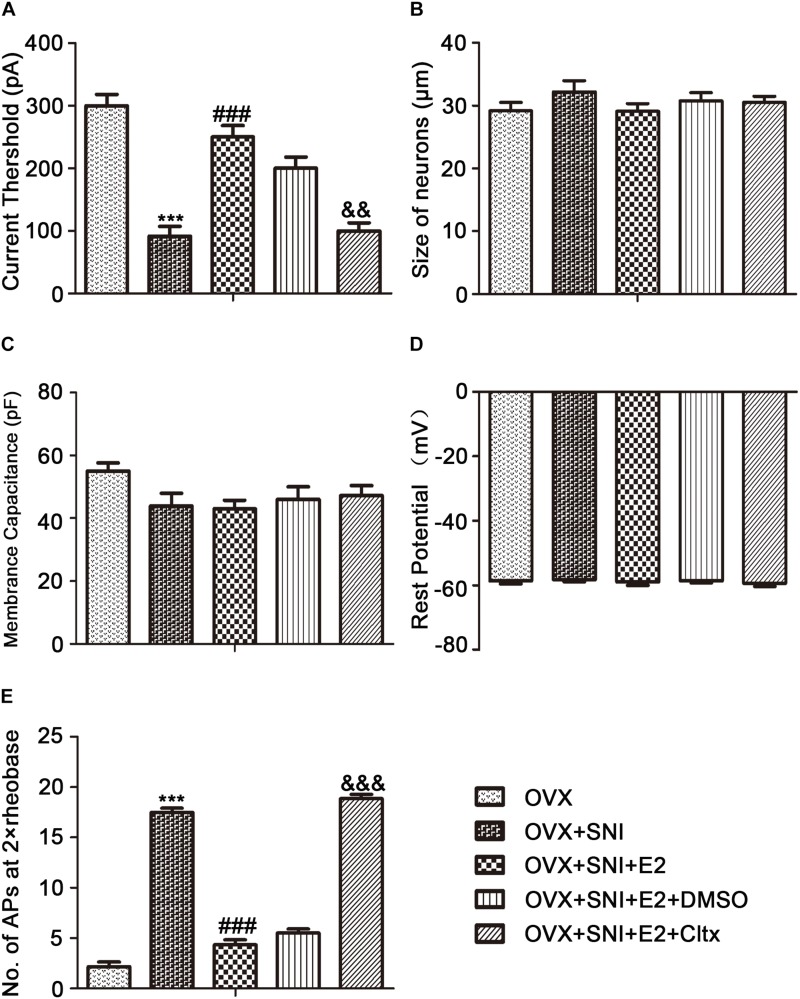
17β-estradiol reversed current threshold (rheobase) decrease and the number of action potentials produced at the corresponding 2 × rheobase increase and was blocked by Cltx. **(A)** Statistical analysis revealed the rheobase. **(B)** Size of neurons. **(C)** Membrane capacitance. **(D)** Resting potential. **(E)** Number of action potentials (APs) at 2 × rheobase in DRG neurons. *n* = 6 per group, ^∗∗∗^*P* < 0.001, OVX + SNI vs. OVX group. ^###^*P* < 0.001, OVX + SNI + E2 vs. OVX + SNI group; ^&⁣&^*P* < 0.01,^ &&&^*P* < 0.001 OVX + SNI + E2 + Cltx vs. OVX + SNI + E2 group.

To examine why 17β-estradiol decreased the excitability for cold sensitivity caused by SNI in OVX rats, we examined the characteristics of the APs of DRG neurons. APs were elicited by a series of depolarizing currents from 0 to 500 pA (150 ms) in 50-pA step increments under the current clamp mode to measure the current threshold (rheobase), i.e., the minimal current that evoked an action potential, which was used as a parameter for excitability (Figures 7A–F). All DRG neurons from OVX rats were harvested on day 10 of SNI, with or without 17β-estradiol administration; DRGs for patch clamps were incubated with Cltx *in vitro*. The data suggested increased excitability of DRG neurons after SNI. Similarly, the voltage threshold of the APs in the OVX + SNI group was significantly lower than that in the OVX group. 17β-estradiol decreased excitability as it was blocked by Cltx (Figure 8A; OVX + SNI group vs. OVX group, 91.67 ± 15.37 vs. 300 ± 18.26, *P* < 0.001; OVX + SNI + E2 group vs. OVX + SNI group, 250 ± 18.26 vs. 91.67 ± 15.37, *P* < 0.001; OVX + SNI + E2 + Cltx group vs. OVX + SNI + E2 group, 100 ± 12.91 vs. 250 ± 18.26, *P* < 0.01; *n* = 6 in each group). The mean number of APs at double-strength rheobase (2 rheobase) was higher in the OVX + SNI group (Figure 8B). When 17β-estradiol was administered, the number of APs decreased under double-strength rheobase stimulation, and increased after intrathecal Cltx administration (Figure 8E; OVX + SNI group vs. OVX group, 17.5 ± 0.4282 vs. 2.167 ± 0.4773, *P* < 0.001; OVX + SNI + E2 group vs. OVX + SNI group, 4.333 ± 0.4944 vs. 17.5 ± 0.4282, *P* < 0.001; OVX + SNI + E2 + Cltx group vs. OVX + SNI + E2 group, 18.83 ± 0.4773 vs. 4.333 ± 0.4944, *P* < 0.01; *n* = 6 in each group). Other action potential parameters such as membrane capacitance, resting membrane potential, and magnitude of APs were not significantly different between the groups (Figures 8C,D). Furthermore, the size of all neurons was between 20–35 μm (Figure 8B). Administration of control solution had no effect on the rheobase and APs.

## Discussion

This study reported that ClC-3 expression in DRG neurons was not significantly changed 2 weeks after OVX. However, according to the literature, mechanical pain was observed 5 weeks after simple OVX and there were also observed changes in pain-related proteins (Amandusson and Blomqvist, 2013; Jiang et al., 2017). We can confirm that OVX has no effect on ClC-3 expression before SNI in this study. ClC-3 is distributed in the central nervous system (Riazanski et al., 2011) and, in this study, its expression decreased following SNI in OVX rats. Notably, ClC-3 was expressed at high levels in DRG cells, especially in medium/small-sized neurons. It was reported that in C57BL/6J mouse DRG neurons, ClC-3 is expressed at a high level especially in small size neurons (Pang et al., 2016). An SNI model was established 2 weeks after OVX; the induced neuropathic pain tended to begin on the 3rd day of SNI and to persist until the 21st day. It was reported that, in male rats, SNI-caused neuropathic pain lasted longer (Vacca et al., 2016). This indicates that OVX may affect SNI-induced neuropathic pain to some degree. However, hyperalgesia and decreased ClC-3 expression in OVX SNI-treated rats were reversed by 17β-estradiol replacement.

Neuropathic pain is a worldwide health concern with poor treatment outcomes (Norcini et al., 2016; Mo et al., 2018; Ouyang et al., 2019). Increases in spontaneous ectopic discharge in DRG neurons have been shown to play a critical role in neuropathic pain genesis (Mo et al., 2018). Small and medium-sized DRG cells were used for all patch clamp experiments. After the establishment of the SNI model, the reduction in ClC-3 expression decreased the activation rheobase of APs and increased the membrane input resistance in DRG neurons. Therefore, the same current injection induced more APs in the DRG neurons of the OVX + SNI group. Decreased ClC-3 expression did not affect cell membrane capacitance, resting membrane potential, or the amplitude of APs in DRG neurons. These findings indicate that increase in the excitability of DRG neurons contributes to hypersensitivity of primary afferent neurons to cold stimulation in OVX + SNI rats.

**FIGURE 9:**
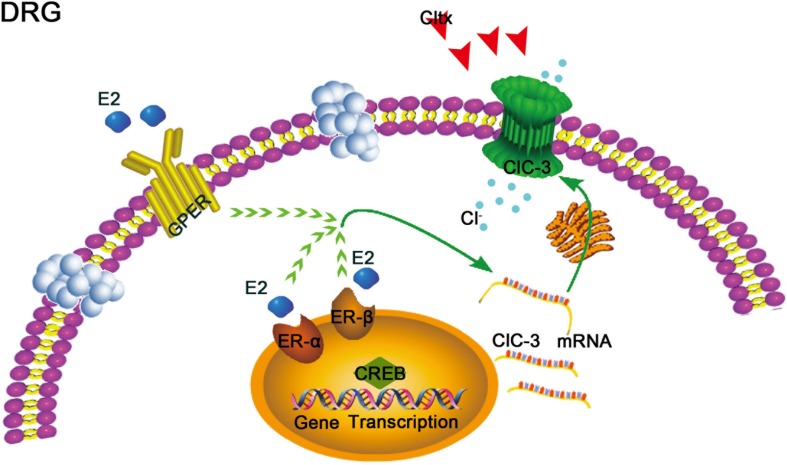
Schematic of potential 17β-estradiol-mediated mechanisms of neuropathic pain regulation in ovariectomized female rats. 17β-estradiol may regulate the expression of *ClC-3* mRNA through the 17β-estradiol receptor, including nuclear receptors α and β or membrane receptor GPER, via certain signaling pathways in the cell, thereby affecting the expression of *ClC-3* mRNA and protein and relieving neuropathic pain. However, this effect may be blocked by Cltx, a specific blocker that affects the function of ClC-3 channel protein but does not affect the expression of mRNA. GPER, G protein-coupled 17β-estradiol receptor. ER-α and ER-β: nuclear 17β-estradiol receptors α and β.

When 17β-estradiol was administered, the increase in excitability was attenuated. Conversely, excitability increased after administration of both 17β-estradiol and Cltx, a ClC-3 specific blocker. The most likely ion channel internalization by Cltx in gliomas is ClC-3 (Thompson and Sontheimer, 2016). Cltx, which binds to ClC-3 with MMP-2/MT1-MMP, forms a macromolecular protein complex on the cell membrane surface that indirectly affects the action of the chloride channel (Deshane et al., 2003; Thompson and Sontheimer, 2016). It differs from NPPB in inhibiting ClC-3 ion channels, as NPPB blocks the function of the ClC-3 ion channel, while Cltx reduces the number of functional chloride channels on the cell membrane surface. Regardless, Cltx was found to cause internalization of ClC-3 into caveolar rafts 15 min after its application (McFerrin and Sontheimer, 2006; Thompson and Sontheimer, 2016; Wang D. et al., 2017). In this regard, 17β-estradiol upregulated ClC-3 in DRG neurons of SNI-model rats at both the gene and protein levels; however, after 17β-estradiol and Cltx were administered, *ClC-3* mRNA levels were not significantly decreased compared to those in the 17β-estradiol-administered group (Figure 9). This suggests that 17β-estradiol may affect the expression of ClC-3 at the gene level, thus increasing the sensitivity to cold stimulation by affecting the excitability of DRG neurons. Interestingly, when Cltx was used in the control OVX group, there were observed behavioral changes in cold allergy, and the allergic reaction increased. That was further verified that estrogen likely regulates neuropathic pain in OVX rats through ClC-3. It is valuable to note that in OVX + SNI rats, Cltx showed limited effects on ClC-3 protein expression and hyperalgesia, this phenomenon indicates that there are other regulatory mechanisms to be studied. The existing literature on the role of 17β-estradiol is inconsistent; both nociceptive and anti-nociceptive 17β-estradiol effects have been reported (Vacca et al., 2016; Sorge and Totsch, 2017; Li W. et al., 2019; Stinson et al., 2019). Furthermore, the results may also depend on 17β-estradiol levels and the structures and systems involved (Craft, 2007; Vacca et al., 2016). Pathological pain can be divided into inflammatory, cancer, and neuropathic (Amandusson and Blomqvist, 2013). Evidence suggests that 17β-estradiol may promote inflammatory pain but has a therapeutic effect on sexual pain (Ma et al., 2016; Vacca et al., 2016); it can also alleviate neuropathic pain caused by chemotherapy through different ERs (Ma et al., 2016; Kramer et al., 2018). Many studies have previously reported that 17β-estradiol can regulate the expression of pain-related proteins in the central nervous system and peripheral neurons such as DRG cells, thereby alleviating SNI-induced neuropathic pain and associated anxiety (Lu et al., 2013; Small et al., 2013; Liu et al., 2015; Ramirez-Barrantes et al., 2016; Vacca et al., 2016; Xu et al., 2017; Lee et al., 2018). Further, 17β-estradiol reduces pain thresholds in neuropathic rats by increasing the expression of NMDAR1 (Deng C. et al., 2017). The pathogenesis of neuropathic pain is mainly underpinned by changes in ion channels that influence APs (Scholz et al., 2019). A previous study reported that altered activity resulted in changes in the properties and/or expression of various types of ion channels, such as voltage-gated Na^+^, K^+^, and Ca^2+^ channels (Waxman and Zamponi, 2014; Daou et al., 2016); however, the role of anion channels remains unclear.

A recent report indicated that ClC-3 is a member of the voltage-gated chloride channel family; its deletion caused increased excitability of DRG cells and decrease in the mechanical pain threshold in rats and mice (Pang et al., 2016). ClC-3 belongs to the ClC voltage-gated chloride channel Superfamily and includes two different functional groups: voltage-gated chloride channels and Cl^–^/H^+^ reverse transporters (Deshane et al., 2003; Riazanski et al., 2011; Liu et al., 2013; Hong et al., 2015). According to previous reports, estrogen may alleviate neuropathic pain (Vacca et al., 2016; Lee et al., 2018). It has been reported that estrogen reduces the pain threshold in males, likely due to its sexually dimorphic actions (Alabas et al., 2012; Bereiter et al., 2019). In neutered females, estrogen has analgesic effects that may be mediated by ClC-3. Previous investigations reported that pain involves two effects that may occur at different times. There are no reports of estrogen increasing pain sensitivity; however, when estrogen levels increase during pregnancy, pain sensitivity is known to decrease, and oophorectomy results in hyperalgesia in mice subjected to mechanical and thermal tests (Amandusson and Blomqvist, 2013; Berman et al., 2017; Ren et al., 2018; Yousuf et al., 2019). However, more studies have favored the antagonistic effect of estrogen on pain (Gintzler and Liu, 2012; Bálint et al., 2016; Kramer et al., 2018). Future studies, performed with OVX female rats or mice, should investigate the role of ClC-3 in the 17β-estradiol-mediated effects on SNI-induced neuropathic pain in OVX animals. These investigations will provide more evidence for the multifarious effects of estrogen on pain. Indeed, this study did not assess compensatory mechanisms caused by dysfunctional hormonal conditions. ERs are widely distributed in the nervous system (Tang et al., 2014; Bálint et al., 2016; Nourbakhsh et al., 2018; Li L. et al., 2019; Liu et al., 2019). Reportedly, estrogen could influence the expression of P2X3 × 3 via ERα and GPR30 to affect neuropathic pain, which may be mediated through the ERK pathway (Lu et al., 2013). Future studies may confirm the mechanisms by which ClC-3 regulates ERs. The results of this study provide a new direction for new treatments in the clinical treatment of neuropathic pain in menopausal women.

## Conclusion

In conclusion, our results showed the complex interactions involved in estrogen-induced pain regulation and revealed the potent role of 17β-estradiol in neuropathic pain, which was altered in female OVX rats. Estrogen may decrease sensitivity to cold stimulation through increased ClC-3 expression in rats experiencing chronic neuropathic pain 2 weeks after OVX.

## Data Availability Statement

The datasets generated for this study are available on request to the corresponding author.

## Ethics Statement

The animal study was reviewed and approved by Institutional Animal Care and Use Committee of the Medical College of Shihezi University.

## Author Contributions

J-QS, Z-ZX, and L-CZ conceived and designed the experiments. Z-ZX conducted the experiments. Q-YC, S-YD, MZ, and C-YT helped with the experiments. Z-ZX and YW analyzed the data. Z-ZX and J-QS wrote the manuscript. All authors discussed and commented on the manuscript.

## Conflict of Interest

The authors declare that the research was conducted in the absence of any commercial or financial relationships that could be construed as a potential conflict of interest.

## Supplementary Material

FIGURE S1 Cltx treat on OVX and OVX + SNI ClC-3 expression experiment 1.

FIGURE S2 Cltx treat on OVX and OVX + SNI ClC-3 expression experiment 2.

FIGURE S3 Cltx treat on OVX and OVX + SNI ClC-3 expression experiment 3.

FIGURE S4 Cltx treat on OVX and OVX + SNI β-action expression experiment 1.

FIGURE S5 Cltx treat on OVX and OVX + SNI β-action expression experiment 2.

FIGURE S6 Cltx treat on OVX and OVX + SNI β-action expression experiment 3.

FIGURE S7 ClC-3 expression after SNI treatment, experiment 1.

FIGURE S8 ClC-3 expression after SNI treatment, experiment 2.

FIGURE S9 ClC-3 expression after SNI treatment, experiment 3.

FIGURE S10 β-action expression after SNI treatment, experiment 1.

FIGURE S11 β-action expression after SNI treatment, experiment 2.

FIGURE S12 β-action expression after SNI treatment, experiment 3.

TABLE S1 Development of cold hypersensitivity after SNI treatment, E2, Cltx treatment.

TABLE S2 Cltx treat on OVX and OVX + SNI cold hypersensitivity development.

## Abbreviations

ClC-3: chloride channel-3
DMSO: dimethyl sulfoxide
DRG: dorsal root ganglion
E2: estradiol
ER: estrogen receptor
OVX: ovariectomized
PBS: phosphate-buffered saline
PWCL: paw withdrawal cold latency
PWTL: paw withdrawal thermal latency
ShamOVX: sham ovariectomized
SNI: spared nerve injury.
