# Supplementary figures and images for: 17β-Estradiol Attenuates Neuropathic Pain Caused by Spared Nerve Injury by Upregulating CIC-3 in the Dorsal Root Ganglion of Ovariectomized Rats
Source: Front Neurosci. 2019 Nov 8;13:1205. doi: 10.3389/fnins.2019.01205 (PMC6856564; doi:10.3389/fnins.2019.01205)

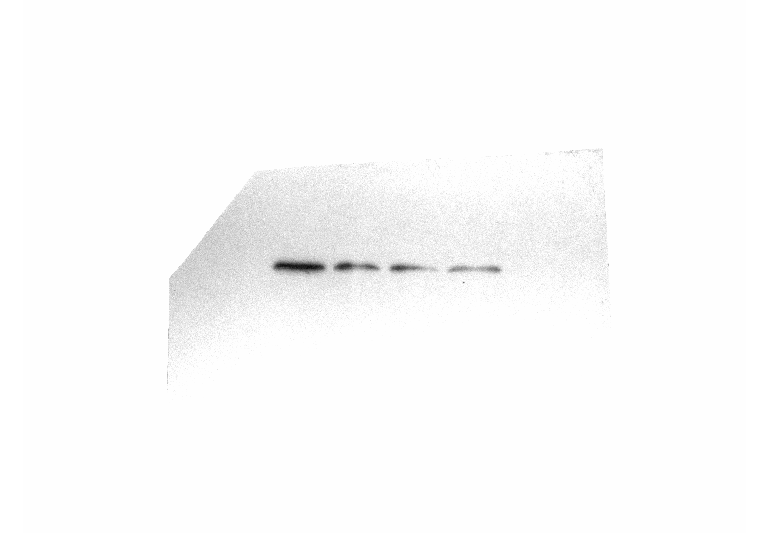

Supplement: FIGURE S1 — Cltx treat on OVX and OVX + SNI ClC-3 expression experiment 1. [file Image_1.TIF]

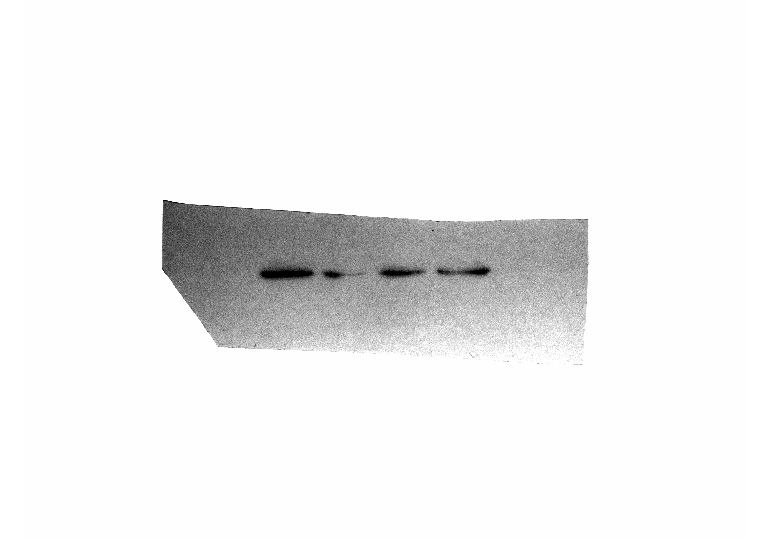

Supplement: FIGURE S2 — Cltx treat on OVX and OVX + SNI ClC-3 expression experiment 2. [file Image_2.TIF]

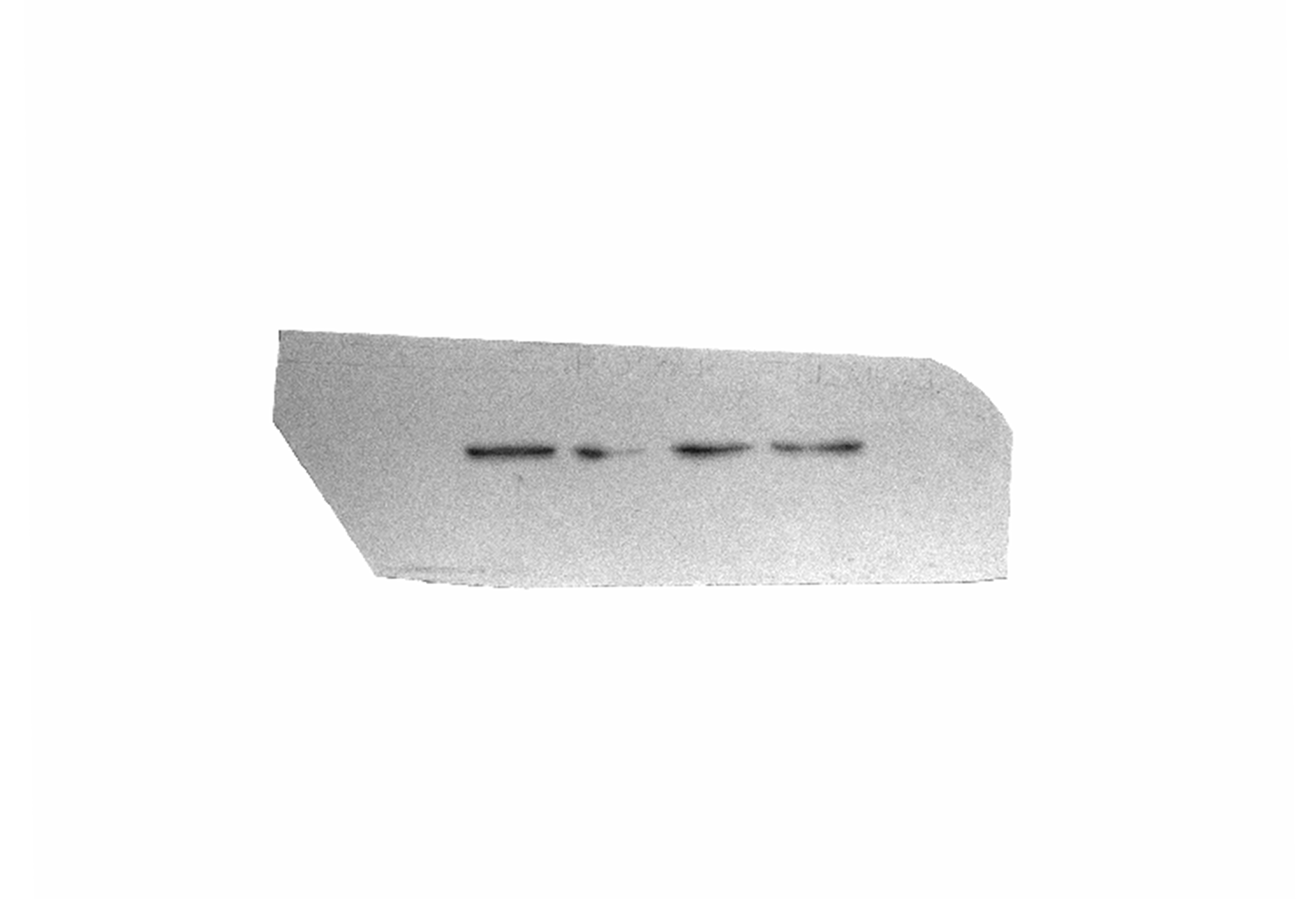

Supplement: FIGURE S3 — Cltx treat on OVX and OVX + SNI ClC-3 expression experiment 3. [file Image_3.TIF]

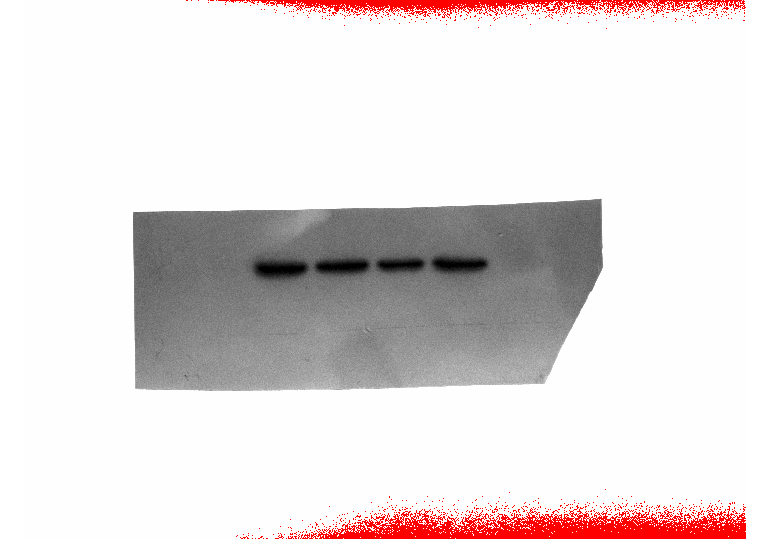

Supplement: FIGURE S4 — Cltx treat on OVX and OVX + SNI β-action expression experiment 1. [file Image_4.TIF]

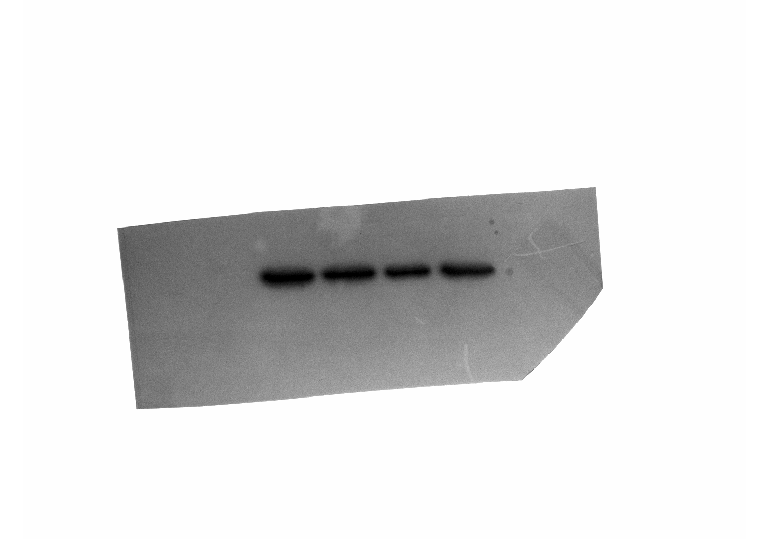

Supplement: FIGURE S5 — Cltx treat on OVX and OVX + SNI β-action expression experiment 2. [file Image_5.TIF]

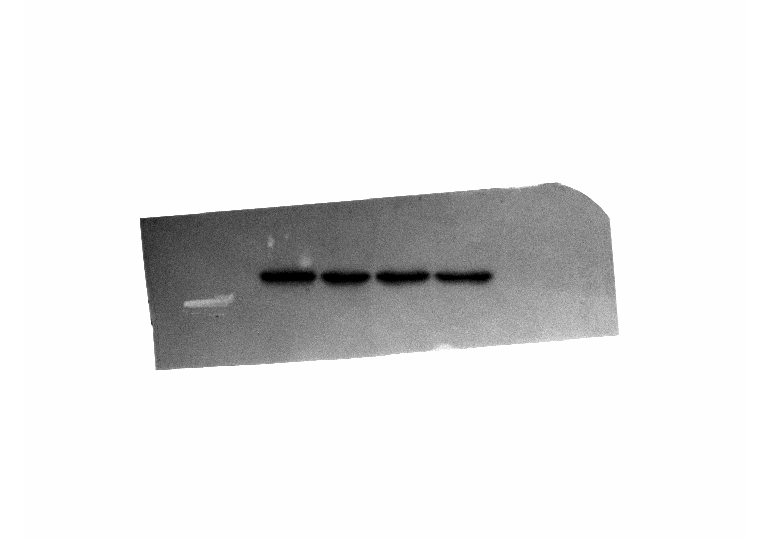

Supplement: FIGURE S6 — Cltx treat on OVX and OVX + SNI β-action expression experiment 3. [file Image_6.TIF]

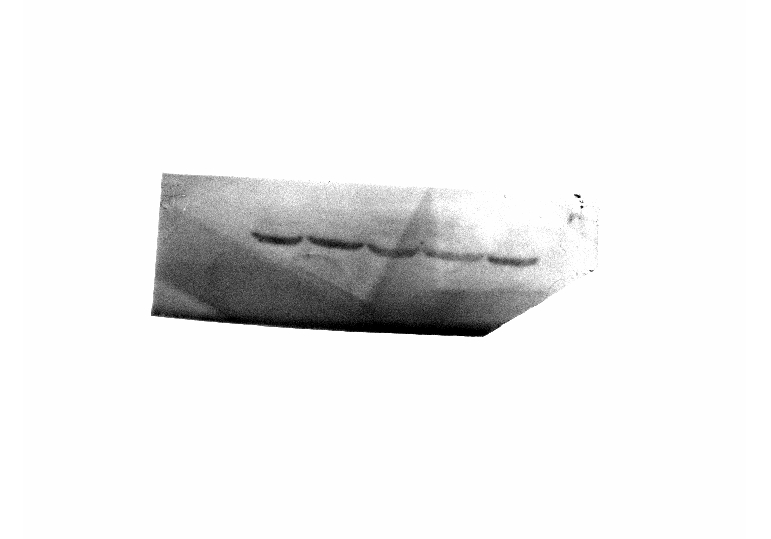

Supplement: FIGURE S7 — ClC-3 expression after SNI treatment, experiment 1. [file Image_7.JPEG]

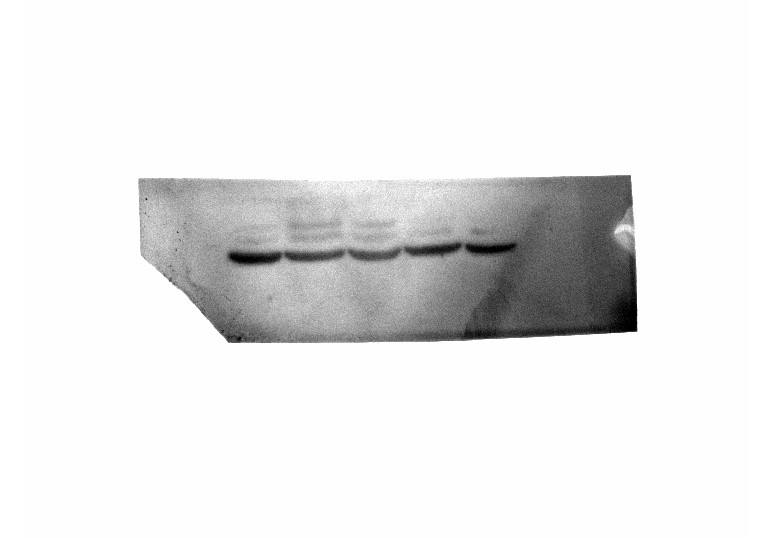

Supplement: FIGURE S8 — ClC-3 expression after SNI treatment, experiment 2. [file Image_8.JPEG]

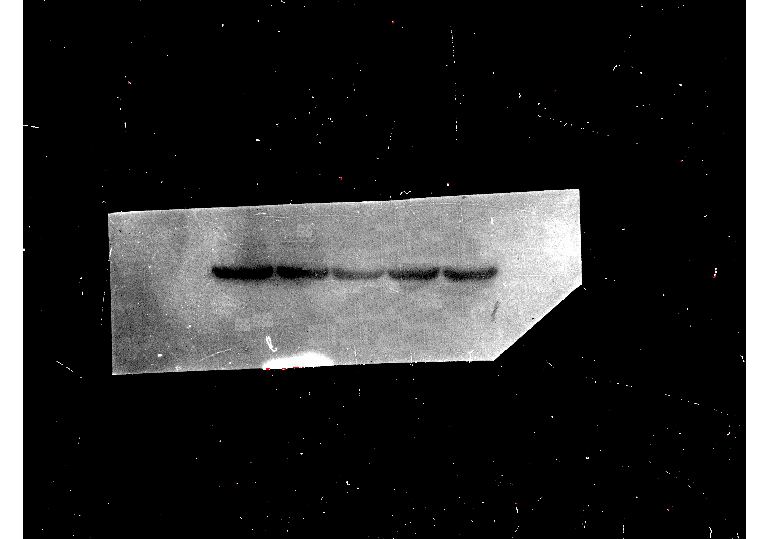

Supplement: FIGURE S9 — ClC-3 expression after SNI treatment, experiment 3. [file Image_9.JPEG]

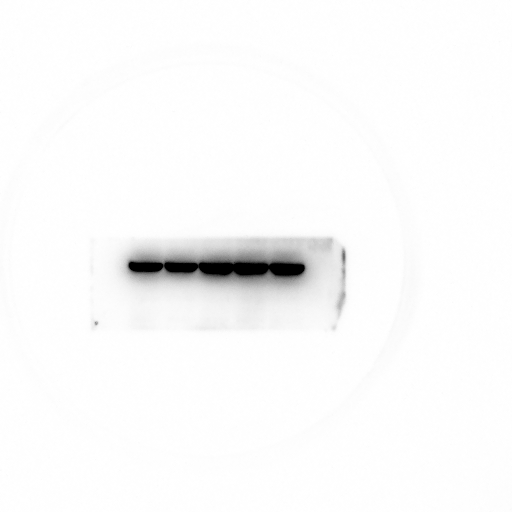

Supplement: FIGURE S10 — β-action expression after SNI treatment, experiment 1. [file Image_10.TIF]

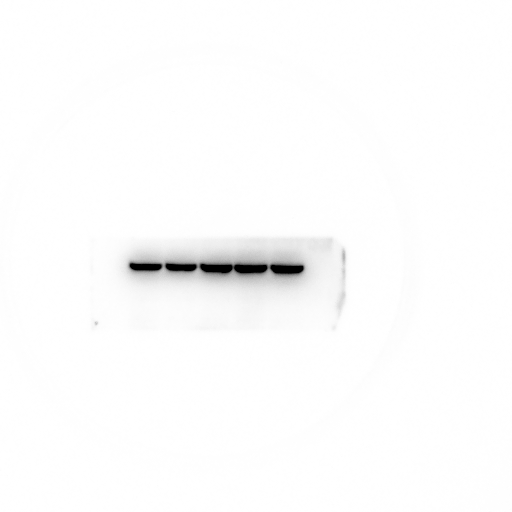

Supplement: FIGURE S11 — β-action expression after SNI treatment, experiment 2. [file Image_11.TIF]

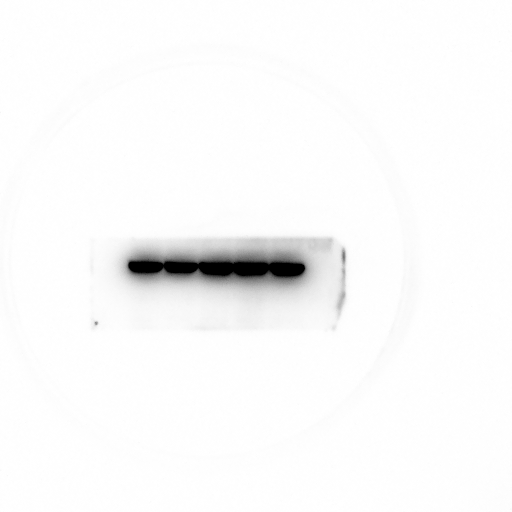

Supplement: FIGURE S12 — β-action expression after SNI treatment, experiment 3. [file Image_12.TIF]
